# A Classification Method for Seed Viability Assessment with Infrared Thermography

**DOI:** 10.3390/s17040845

**Published:** 2017-04-12

**Authors:** Sen Men, Lei Yan, Jiaxin Liu, Hua Qian, Qinjuan Luo

**Affiliations:** School of Technology, Beijing Forestry University, Beijing 100083, China; mensen1989@163.com (S.M.); liujiaxin@bjfu.edu.cn (J.L.); qianhua@bjfu.edu.cn (H.Q.); luoqinjuan@bjfu.edu.cn (Q.L.)

**Keywords:** thermal imaging, support vector machine (SVM), seed germination, multi classifier, image processing, classification

## Abstract

This paper presents a viability assessment method for *Pisum sativum L.* seeds based on the infrared thermography technique. In this work, different artificial treatments were conducted to prepare seeds samples with different viability. Thermal images and visible images were recorded every five minutes during the standard five day germination test. After the test, the root length of each sample was measured, which can be used as the viability index of that seed. Each individual seed area in the visible images was segmented with an edge detection method, and the average temperature of the corresponding area in the infrared images was calculated as the representative temperature for this seed at that time. The temperature curve of each seed during germination was plotted. Thirteen characteristic parameters extracted from the temperature curve were analyzed to show the difference of the temperature fluctuations between the seeds samples with different viability. With above parameters, support vector machine (SVM) was used to classify the seed samples into three categories: viable, aged and dead according to the root length, the classification accuracy rate was 95%. On this basis, with the temperature data of only the first three hours during the germination, another SVM model was proposed to classify the seed samples, and the accuracy rate was about 91.67%. From these experimental results, it can be seen that infrared thermography can be applied for the prediction of seed viability, based on the SVM algorithm.

## 1. Introduction

Seed viability assessment is a key component of agricultural production and commercialization. Seed viability can be affected by several factors, including overheating, physical damage and natural aging. Assurances of high seed productivity are necessary for seed users in agricultural production, and meanwhile high seed viability needs to be guaranteed in agricultural commercialization to ensure the business optimization of seed companies. Hence, both the seed users and seed supply companies are required to invest in seed viability test and classification technologies.

In seed viability assessment, many conventional methods including the standard germination test, electrical conductivity test, seedling growth test, accelerated aging test and triphenyltetrazolium chloride (TTC) quantitative analysis have been proposed [1,2]. However, several shortcomings still exist in these methods, such as invasiveness, huge amount of test work needed, long test periods, low accuracy and obvious subjective effects [3]. Therefore, fast and nondestructive diagnosis methods are urgently required in seed viability assessment. In view of this, several optical techniques, such as infrared thermography, Fourier transform infrared, Fourier transform near-infrared, bio-speckle, nuclear magnetic resonance, ultraviolet-visible and Raman spectroscopy and hyperspectral imaging have been developed to estimate the seed viability [4,5,6,7,8,9,10]. 

Although these methods have the merits of being non-contact and providing rapid inspection, they also have limitations, such as the high cost of hyperspectral imaging and nuclear magnetic resonance devices, low throughput in Fourier transform infrared, Fourier transform near-infrared and ultraviolet-visible spectroscopy, Raman spectroscopy and bio-speckle. By contrast, infrared thermography shows the advantages of relatively low cost and high assessment throughput, so considering all the factors, infrared thermography is a proper technique in seed viability assessment [11,12,13,14,15].

Recently, many researchers have paid special attention to the application of infrared thermography. As a noninvasive diagnosis method, infrared thermography has been applied in various fields including medical tests, fault diagnosis, remote sensing, plant diseases and insect pest detection, fruit quality and seed viability assessment [13,14,15]. In these applications, seed viability estimation with infrared thermography is what we are most concerned about. In previous studies, the variation of seed heat flow has been proved to be affected by water imbibition, respiration, decomposition of nutrients and other biochemical, physical, chemical reactions correlated with seed viability during the germination processes [16,17]. The surface of seeds measured by infrared thermal cameras can be recorded as the temperature data for non-destructive seed viability evaluation. Hence, the infrared thermography technique has great potential in seed quality assessment.

Microcalorimetry techniques have been used to depict the variation of seed heat flow and it is proved that gross metabolism is associated with germination processes [18,19,20]. However, a closed system is needed in microcalorimeter measurements to prevent the dissipation of heat and gas. This closed system can induce the perturbation of potential and lead to confounding seed metabolism feedback. In comparison, infrared thermography can capture thermal activity in the phase of seed imbibition thanks to its large-area scanning advantage. This technique was first used to evaluate the germination capacity of leguminous seeds in 2003, and the results demonstrated that temperature changes in seeds with germination showed a considerable decrease in radiation temperature (more than 1 °C) during the first 12 h [21]. Further studies about seed viability assessment with the infrared thermography technique found that whether seeds would germinate or not could be predicted in the first three hours of the water uptake period, when the seeds could be redried and stored again [22]. 

In seed viability detection with infrared thermography, a temperature-time curve for every seed is generally plotted by using temperature values in time sequence in the acquired thermal images. As the main classification method in previous studies, the minimum distance algorithm is widely adopted to evaluate the seed viability. However, this algorithm is easily affected by the selection of experimental samples. In terms of classification problems, many intelligent algorithms including decision trees, Bayesian classification, KNN (K-nearest neighbors) and artificial neural net were introduced [23]. Based on the statistical theory, these classification methods need quite a large amount of samples that cannot be satisfied in practical problems. Compared with these methods, the target of support vector machine (SVM) is structural risk minimization instead of empirical risk minimization [24,25]. Therefore, the SVM method has the advantages of global optimization and generalization ability, which is suitable for seed viability assessment where a small amount of samples are classified with infrared thermography.

As a new technique in seed viability assessment, it is important to investigate the relationship between the seed heat flows detected by infrared thermography and the seed viability. Considering its non-destructiveness, the infrared thermography technique can be used during the whole sample germination process. It can detect the seed viability during the period of water uptake and improve the accuracy of viable seed assessment by virtue of the temperature change curves of thermography images. 

The objectives of the present study included: (1) investigation of the differences in temperature curves of highly viable, aged and dead seeds during the germination period; (2) exploration of the vital parameters of temperature curves; (3) development of a method that can distinguish the viable seeds and unviable seeds before germination.

## 2. Materials and Methods 

### 2.1. Plant Materials

*Pisum sativum* L. seeds were selected as the experimental samples, and submitted to standard germination tests for quality control. A total of 120 seeds were divided into two groups, namely A class and B class, for different treatments (80 seeds for A class and 40 seeds for B class). The seeds of class A were stored at 5 °C for three days while the seeds of class B were treated at 100 °C for three days. To be specific, A class seeds were put in a refrigerator at 5 °C for three days, while B class seeds were treated at 100 °C (±1 °C) with a draught drying cabinet. Root lengths of each individual seed were then measured by a Vernier caliper on the fifth day after imbibition so as to qualify the seed viability. 

**Figure 1:**
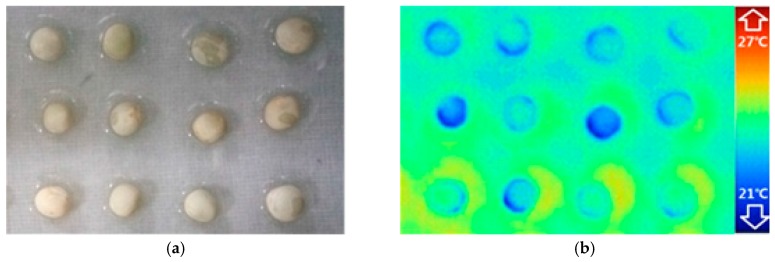
Visible (**a**) and thermal images (**b**) of the experimental samples.

Polycarbonate plates (cryogenic vial holders with holes) were used as the Petri dish in the germination experiment. The polycarbonate plate was placed in a water bath and covered with filter paper. Every seed was placed above the well of the polycarbonate plate with a little well-hydrated dip formed under the seeds. Ambient temperature, including both air and water temperature, was maintained constant at 24 °C with minimal convection to reduce the environment impact on seeds. The infrared thermography system, including a light source, infrared thermal camera, charge coupled device (CCD) and waterbath was put in the constant temperature incubator. The visible and thermal images that captured by infrared camera and CCD respectively, are shown in Figure 1. During the standard germination test, the temperature incubator has held constant at 24 °C (±0.4 °C) and the seeds continuously exposed to light till the end of the experiment. 

### 2.2. Image Analysis

**Figure 2:**
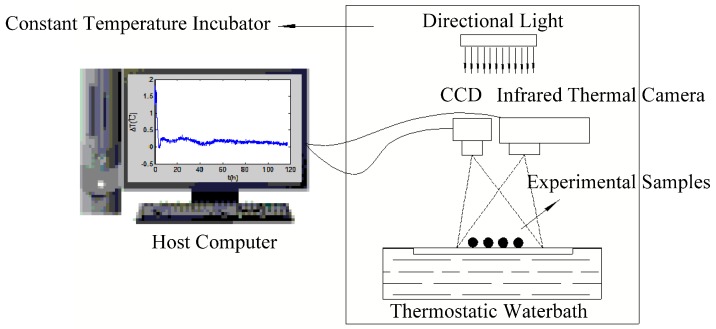
Schematic diagram of infrared thermography system used to capture and analyze thermal profiles.

Figure 2 shows a schematic of the infrared thermography system used to capture and analyze the thermal and visible images of the seeds. This system consists of an infrared thermal camera, a digital color charge-coupled device (CCD) camera, a directional light source, a constant temperature incubator, a thermostatic waterbath, and a host computer. A resolution of 320 × 240 pixels thermal images were registered by the infrared thermal camera Ti55 (Fluke, Everett, WA, USA) with a sensitivity of 0.02 °C and preliminarily processed with the Smartview software (Fluke Systems). Visible images with a resolution of 900 × 600 pixels acquired from the CCD were digitized to 8 bit (256 grey levels) data and stored. Both thermal and visible images were stored every 5 mins over five days and could be exported as individual images or as a series of images in time sequence. Afterwards, these thermal and visible images were analyzed with the software MATLAB (MathWorks, Natick, MA, USA) for post-processing.

**Figure 3:**
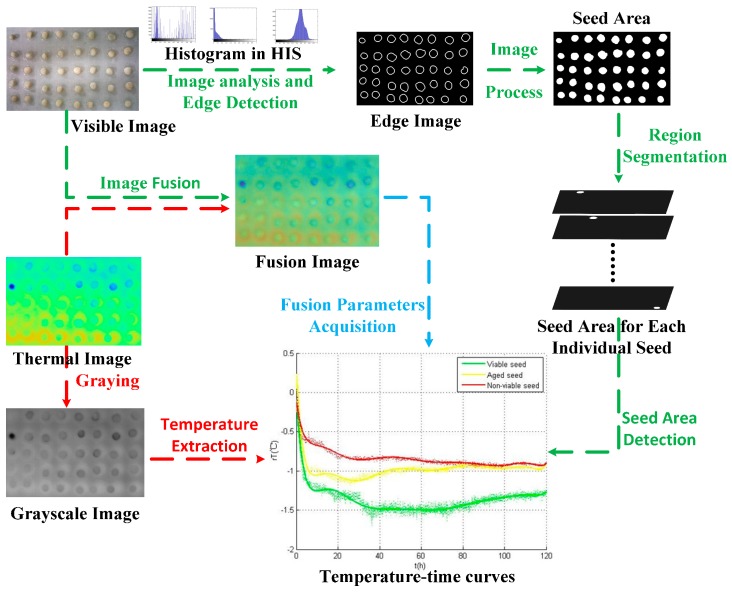
Flow chart of a series of steps for analyzing thermal and visible images data. Visible images are used to acquire the edge regions information (green arrow). Thermal images are used to extract the temperature information (red arrow). Fusion parameters are acquired from fusion image of viable image and thermography image.

As shown in Figure 3, due to the effect of water uptake and respiration, heat convection between the seeds and the ambient (both air and water) lasts during the whole germination period until the end of the experiment. Under the impact of this convection, the edges of the seeds in the profiles merge into the background, but in the visible images, the unabridged edges can be detected. By the image fusion technology, the temperature information of seed areas restricted by the shapes in the visible images could be acquired to plot the curves of seeds in the different viability categories during the germination period.

Regarding the characteristics of visible images, we propose an image processing flow to acquire each individual seed areas in the images. As a pre-processing approach, background subtraction can eliminate any non-uniform brightness areas and reflection points in the visible images. The edges of individual seeds were detected by the edge extraction method, and a region growing method was introduced to form the segmented regions. A disk whose area was approximately one-third of the seed area was placed in the center of each seed and defined as the seed area.

Thermal images obtained during the experiment were displayed as pseudo-color images and were transformed into grayscale images which recorded the temperature as gray values. Maximum (grey level is 255) and minimum (grey level is 0) gray values corresponded respectively to the preset maximum (27 °C) and minimum (21 °C) temperature value before the experiment.

Image fusion technology was adopted to extract seed regions in the thermal images with the disk areas in the visible images. Then, the temperature data of each individual seed was obtained through multiplication of thermal images and visible images. The average over all pixels of the seed region was calculated as the resulting temperature value *T* and the value can be expressed as the follow equation (Equation (1)):

(1) $$T=\sum_{i=1}^{n}\left[I_{i}/n\times (T_{max}-T_{min})/(255-0)\right]$$

where *I_i_* is the grey value of the *i* pixel in the thermal image, *n* represents the total pixel number of the seed region. *T_max_* and *T_min_* are the pre-set maximum and minimum temperature values. 255 and 0 are the maximum and minimum grey values in the thermal image. 

For the purpose of correcting the temperature value, the temperature of filter paper area around each individual seed area was defined as the environmental temperature of the seed. This ambient area temperature was then introduced into the calculation. Similar to the calculation of temperature value in the seed area, the environment temperature can be calculated by (Equation (1)). Define *rT* as the temperature difference between the seed area and the environment area, namely. This difference was expressed in the equation (Equation (2)):

(2) $$rT=T_{seed}-T_{environment}$$

A total number of 1440 thermal images were used to describe the temperature variation for each individual seed during the experiment. This variation was analyzed by the software MATLAB to obtain the temperature curve of each individual seed.

The temperature-time curve of each seed was analysis to extract its characteristic parameters. Then, these parameters were measured by Least Significant Difference (LSD) multiple comparison analysis method. LSD method is used to analyze the multiple comparisons as follows: calculate the ratio of absolute temperature value of two variables $\left|{\bar{x}}_{i}-{\bar{x}}_{j}\right|$ and its standard error of mean difference. The standard error of the mean difference was calculated using the following equation (Equation (3)):

(3) $$S_{{\bar{x}}_{i}-{\bar{x}}_{j}}=\sqrt{2MS_{e}/n}$$

where *MS_e_* is mean square error in F test, *n* is number of variables.

The ratio would be compared with the critical value of 2-tailed samples T-test (*α* = 0.05). The equation *LSD_α_* could be transformed into the equation (Equation (4)) as:

(4) $$LSD_{\alpha }=t_{0.05}(df_{e})$$

The results shows that ${\bar{x}}_{i}$ and ${\bar{x}}_{j}$ have a significant difference in *α* level if the ratio is higher than *LSD_α_*. Conversely, the difference between ${\bar{x}}_{i}$ and ${\bar{x}}_{j}$ is not significant.

### 2.3. Classification Algorithm

Support vector machine (SVM) was first introduced by Cortes as a universal feed-forward classification algorithm. The SVM approach is a supervised method based on the statistical learning theory and structural risk minimization principle, and can be used to analyze data and recognize images. The strategy of SVM classification is to find an optimal separating hyperplane between classes by focusing on training samples that locate at the edge of the class distributions. The main characteristics of SVM are as follows:

(1) SVM can be generalized in high-dimensional spaces with only a small amount of training samples.
(2) The optimum result can be given by SVM through transforming the problem into a quadratic programming problem.
(3) SVM can simulate nonlinear functional relationships.

A brief description of the SVM classification is given below. In a binary classification problem, the aim is to develop a classifier that generalizes accurately for predicting the membership of a class *y_i_* (−1, +1) from *m*-dimensional input data represented by a vector *X* = {*x*_1_, *x*_2_, *…*, *x_m_*}. In the case of seed viability assessment based on infrared thermography, *m* represents the number of the characters of the temperature variation curve during the germination. Before prediction, it is necessary to train a data set containing the characters corresponding to *n* experimental samples of a known class.

The core of SVM algorithm is to search the optimal hyperplane that separates different classes. This hyperplane can be described as the follow equation (Equation (5)):

(5) $$w\cdot X_{i}+b=0$$

where *w* is the normal vector of the hyperplane and *b* is the offset. During the training process, SVM tries to find the hyperplane that can not only maximize the shortest distance from this hyperplane to the closest training sample of each class (the class *y_i_* = +1 and the class *y_i_* = −1), but also minimize the classification error. The support vectors of the two classes lie on two hyperplanes that are parallel to the optimal hyperplane. The distance between these two planes is defined as the margin associated with the separating hyperplane. The optimization of this margin can be converted into a constrained quadratic optimization problem as follow equation (Equation (6)):

(6) $$\left\{ \begin{array}{l} \min(1/2{\left\|w\right\|}^{2}+C\sum_{i=1}^{n}\xi_{i})\\ s.t.\;\xi_{i}+y_{i}(wX_{i}+b)-1\geq 0\\ \;\xi_{i}\geq 0 \end{array}\right.$$

where *ξi* represents the classification error for the distance between the misclassified sample *i* and the corresponding margin hyperplane and *C* is the regularization meta- parameter controls the trade-off between the two conflicting objectives, i.e., margin maximization and error minimization. When *C* is small, margin maximization is emphasized; whereas when *C* is large, error minimization is predominant. According to the Lagrangian dual formulation, the optimal hyperplane can be expressed as a liner combination of the training observations in the following equation (Equation (7)):

(7) $$\left\{ \begin{array}{l} f(X)=w\cdot X+b=\sum_{i=1}^{n}y_{i}\alpha_{i}\xi_{i}X_{i}+b\\ \alpha_{i}\geq 0 \end{array}\right.$$

where *α_i_* is a Lagrange multiplier that corresponds to a coefficient associated with each object. The magnitude of *α_i_* is related to the parameter *C* and varies between 0 and *C*.

For nonlinear classification problems, the input data are mapped into a high dimensional space through a mapping function. Then the data can be separated with a linear SVM. In the dual representation, a kernel, the inner product of two vectors of *u*(*x*_1_) and *u*(*x*_2_) is used as the mapping function. In this study, the radial basis function (RBF) kernel is used for its advantage of good performance in obtaining almost all boundary shapes. The RBF kernel function is given as the following equation (Equation (8)):

(8) $$K(X_{1},X_{2})=\varphi (X_{1})\cdot \varphi (X_{2})=\exp(-\left\|X_{1}-X_{2}\right\|/\sigma^{2})$$

where *φ*(*X*_1_) and *φ*(*X*_2_) are the mapping functions of the objects *X*_1_ and *X*_2_ respectively; *σ* is the kernel parameter determined by the kernel width meta-parameter.

As a conclusion, the two meta-parameters regularization parameter *C* and kernel parameter *G* need to be selected properly as they determine the boundary complexity and the observed classification rate. 

The multi classifier is integrated by a single classifier with a certain difference. In this work, a multi classifier *y* (viable seeds, aged seeds, non-viable seeds) consisted of three two-class classifiers *y*_1_ (viable seeds, aged seeds) *y*_2_ (viable seeds, non-viable seeds) and *y*_3_ (aged seeds, non-viable seeds). To combine these classifiers, the weighted voting algorithm is adopted. The resultant class is given by choosing the class voted by the majority of the classifiers. Only samples from two classed of each individual classifier are used for training. 

To be specific, all the classifiers were regarded as a voter with a weight value to produce classification results. The performance differences of the base classifiers was introduced by assign a weight value to each base classifier, and the equation (Equation (9)) is shown as follows:

(9) $$\alpha_{i}=p_{i}/\sum_{i=1}^{n}p_{i}$$

where *α_i_* is the weight value of *i* based classifier. *p_i_* represents the average accuracy of the training set of the *i* based classifier.

## 3. Results and Discussion

### 3.1. Viability Test

After the standard germination test, seeds viability was defined as the percentage of germinated seeds and classified by the root lengths. Based on the statistical analysis of five days of germination experiments, the germination rate of A class was 91.25%, whereas the B class seeds did not germinate. According to the root lengths, the seeds (a total of 120 seeds) were classified into three viability categories according to different lengths (Table 1). A class seeds were divided into three viability types, which are viable seeds (Viability type 2), aged seeds (Viability type 1) and non-viable seeds (Viability type 0) and the standards of classifications were explanted in Table 1.

### 3.2. Temperature Variation 

**Figure 4:**
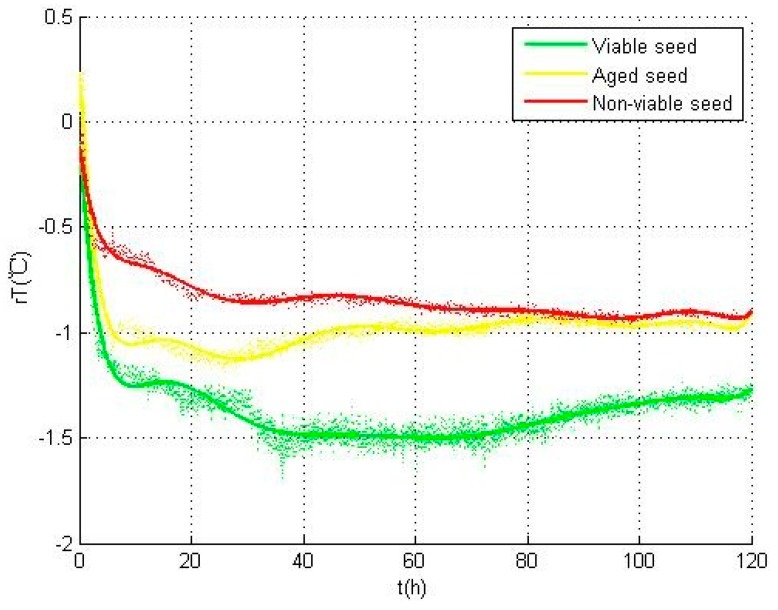
Temperature variations of the seeds for different categories during the experiment.

Figure 4 shows the temperature variations of the seeds temperature in different categories. The three temperature curves were calculated respectively by the averages of viable seeds A2 (green), aged seeds A1 (yellow), and non-viable seeds A0 and B0 (red). In these curves, relative seed temperature (*rT*) acquired by the difference between every individual seed temperature and the environment temperature was used to describe the temperature variations during all the 5 days experiments. The heat variation, i.e., the warming or cooling of the seed, was revealed by the positive or negative of the *rT* value. 

**Figure 5:**
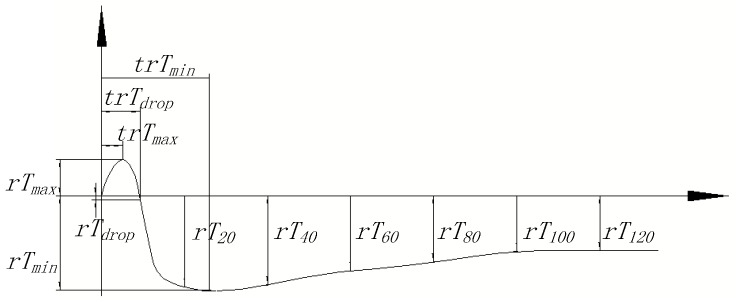
Temperature curve of an individual seed and the definitions of the characteristic parameters.

According to the differences in the three categories of seeds, a curve of an individual seed was illustrated in Figure 5, where several characteristic parameters were marked. Hereinto, *rT_max_* represents the maximum temperature value; *trT_max_* represents the time for reaching the maximum temperature value; *rT_drop_* represents the temperature value at the beginning of its sharp decline; *trT_drop_* represents the time when sharp decline of the temperature begins; *rT_min_* represents the minimum temperature value; *trT_min_* represents the time for reaching the minimum temperature value; *rT*_0*h*_, *rT*_20*h*_, *rT*_40*h*_, *rT*_60*h*_, *rT*_80*h*_, *rT*_100*h*_ and *rT*_120*h*_ represents the temperature values at 0 h, 20 h, 40 h, 60 h, 80 h, 100 h and 120 h, respectively. All these characteristic parameters of the three categories of seeds were statistically calculated and listed in Table 2.

As shown in Figure 4, in viable type A2 seeds (green), *rT* first showed a gentle dip in the first one hour, and then drops sharply within the next two hours till the inflection point to rise up. Due to this phenomenon, the maximum value of temperature (*rT_max_*) occurs at the begining and the minimum value of temperature (*rT_min_*) appears at the inflection point. By contrast, in aged type A1 seeds, *rT* first shows a small peak and reaches the maximum temperature value (*rT_max_*). The sharp decline in this curve was delayed nearly half an hour than that in the curve of viable seeds. The sharp decline appears at the beginning of the curve of the seed of non-viable type A0 and B0 seeds types and reaches the minimum temperature value (*rT_min_*) earlier than that of viable and aged types.

These differences in the temperature curves are mainly reflected in the imbibition period, and can be explained by the biophysical and biochemical changes occurring during the germination test. The imbibition period was affected by the membrane permeability. Compared with viable seeds, the membrane permeability of aged seeds was changed and water absorption in the water uptake period became slower, which resulted in the delay of sharp decline in aged seeds. For non-viable seeds, although the seeds lost the viability, they still had certain water absorbing capacity [22,26]. Considering that different categories of seeds have different characteristics in the internal metabolic activity, the seeds showed distinct differences in their performance. To be specific, the non-viable seeds had the fastest temperature rise in this process, the aged seeds followed, while the viable seeds had the slowest. 

To sum up, considerable variations of cooling in temperature can be observed for all three categories of seeds during the experiments. To prevent the cooling produced by evaporation, relative seed temperature was used to evaluate the cooling. In this situation, the decline of temperature was caused by the biophysical and biochemical changes in seeds instead of evaporation. Multiple comparisons are used to analyze the characteristic parameters in Table 2, and all the parameters with significant difference in three categories of seeds are shown in Table 3. 

**Figure 6:**
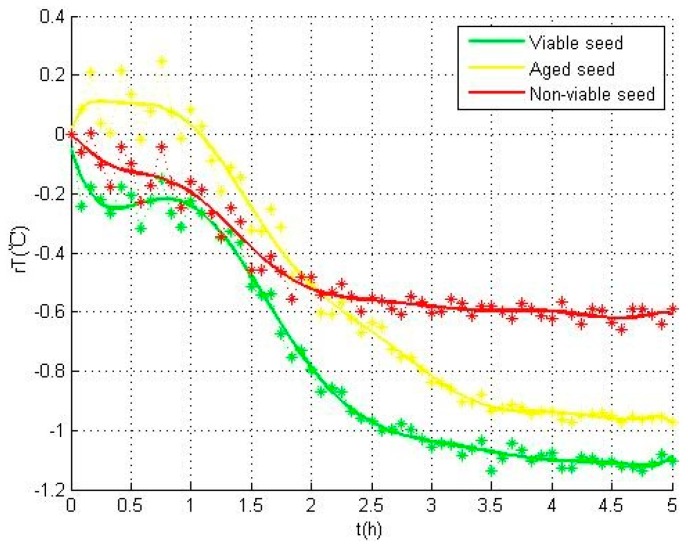
Heat production during the first five hours in the experiment.

As shown in Figure 6, the temperature variations of all the three categories of seeds mainly occur in the first three hours. The parameters including *rT_drop_*, *rT_min_*, and *rT*_0*h*_ can be required from these temperature data, so the temperature data of the first three hours were used for seed viability assessment when the seeds can be redried and stored again. Hence, by virtue of the temperature data in this time period, an SVM model can be developed to assess the seed viability. 

### 3.3. Classification Model

With the analysis, there are significant differences between the above 13 characteristic parameters. These parameters were used as the input of SVM model to explore the classification result with the whole temperature data during the germination. Both the regularization parameter and kernel parameter of this SVM model set to 2. 5-fold cross validation was used in the training of this model and the results of cross validation of the SVM model with the whole germination temperature data is shown in Table 4. 

Based on the analysis and statistics of Table 4, the classification results of the SVM model with the whole germination temperature data is shown in Table 5. As seen in this data, the classification accuracy for all types of seeds is 95%. What’s more, the classification accuracy of each type of seeds can be more than 90%. This result proved that the infrared thermography technique can be used in the viability assessment of pea seeds.

Although the above model proves the accuracy and effectiveness in seed viability assessment with infrared thermography, we still explored the possibility of classification of the different types with less temperature data, especially with the temperature data before the seeds germinate. According to the results in Table 3 and the following analysis, the first three hours of temperature data are selected as the SVM input. These data were represented by the temperature data obtained every ten minute in the first three hours. Both the regularization parameter and kernel parameter of this SVM model set to 2. Five-fold cross validation was used in the training of this model and the results of cross validation of the SVM model with the first three hours temperature data is shown in Table 6. 

Based on the analysis and statistics of Table 6, the classification results of SVM model with the first three hours temperature data is shown in Table 7. As shown in Table 7, the classification accuracy of all types of seeds is 91.67%.

Comparing the SVM model with the whole germination temperature data and the SVM model with the first three hours of temperature data, as can be seen, the overall accuracy rate of the latter for the samples is 91.67%, slightly lower than that of the classification (95%). Hereinto, with the whole germination temperature data, the accuracy rate for the viable seeds is 97.56%; that for the aged seeds is 75%, which is the lowest; that for the non-viable seeds is up to 97.87%, which is the highest. By contrast, with the first three hours temperature data, the accuracy rate for the viable seeds is 92.68%; that for the aged seeds is 90.63% and that for the non-viable seeds is up to 100%. For the comparison results of SVM model, it can be seen that the accuracy rates of viable seeds and non-viable seeds have no significant differences between these two classification models, but the accuracy rate of SVM model with the whole germination temperature data higher than that of SVM model with the first three hours temperature data. 

From analysis and comparison of these two SVM models, it can be concluded that the infrared thermography technique can be used to predict the viability categories of the seeds in the first three hours when seeds can be redried and stored again. The method proposed in this work can be applied for seed viability assessment with the advantages of being fast and nondestructive. 

## 4. Conclusions

In this study, the infrared thermography technique was proposed as a viability assessment method for pea seeds based on their temperature variations. The temperature on the surfaces of experimental samples was measured in a non-destructive and non-contact way by this technique, and the viability of seeds of different categories were obtained by artificial aging. The thermal profiles of the seeds were recorded during the germination experiment, and the temperature curve for each individual seed was plotted by the method of image processing and image fusion. Finally, the SVM model, as a multi-classification method, was used to classify the seeds into viable, aged and non-viable types according to the root length.

The results showed that there are significant differences between the parameters used for characterizing the temperature variations of seeds depended on the seed viability. With these parameters, SVM was used to classify the seed samples into three categories, and the classification accuracy rate was 95%. On this basis, another SVM model was proposed to predict the seed viability in the first three hours when the seeds can be redried and stored again, and result in an overall accuracy rate of 91.67%. 

Our work indicates that the infrared thermography technique can be used as a fast, non-invasive method in seed viability assessment, and has great potential in the viability assessment for various agricultural specimens. In the future, more data from extensive experiments are required to illuminate the relationship between the temperature variations and the specific biophysical and biochemical activities.

## Figures and Tables

**Table 1:** Viability types of the seeds.

Seed Group	Treatment	Root Length (cm)	Seed Viability Type	Seed Amount
**A Class**	5 °C for three days			Total 80
		1.6–5.0	Viable seed (A2)	41
		0.1–1.5	Aged seed (A1)	32
		0	Non-viable seed (A0)	7
**B Class**	100 °C for three days			Total 40
		0	Non-viable seed (B0)	40

**Table 2:** Characteristic parameters of viable, aged and non-viable seeds.

Parameter	Viable Seed Mean ± SD	Aged Seed Mean ± SD	Non-Viable Seed Mean ± SD
*rT_max_*	0.1376 ± 0.2007	0.1838 ± 0.2755	0.1770 ± 0.2858
*trT_max_*	3.8000 ± 2.6099	1.7500 ± 1.7538	1.7000 ± 1.8952
*rT_drop_*	−0.0981 ± 0.3868	−0.1867 ± 0.3801	−0.0248 ± 0.2927
*trT_drop_*	22.7000 ± 4.4919	22.8500 ± 3.7353	15.6000 ± 2.9637
*rT_min_*	−1.0389 ± 0.7089	−1.4796 ± 0.7716	−0.5591 ± 0.9178
*trT_min_*	45.5000 ± 4.7564	48.5500 ± 3.9538	44.0000 ± 4.9593
*rT*_0*h*_	−0.2594 ± 0.5248	0.2288 ± 0.6147	0.0467 ± 0.5589
*rT*_20*h*_	−1.1681 ± 0.6047	−1.3426 ± 0.6570	−0.8700 ± 0.5835
*rT*_40*h*_	−1.3102 ± 0.4897	−1.2525 ± 0.5880	−0.8813 ± 0.5870
*rT*_60*h*_	−1.3474 ± 0.5551	−1.2057 ± 0.5677	−0.8976 ± 0.5751
*rT*_80*h*_	−1.2637 ± 0.5033	−1.1617 ± 0.5428	−0.9496 ± 0.5408
*rT*_100*h*_	−1.1722 ± 0.5064	−1.1788 ± 0.5639	−0.9661 ± 0.5038
*rT*_120*h*_	−1.1240 ± 0.5901	−1.1905 ± 0.6134	−0.9680 ± 0.5092

**Table 3:** Multiple comparisons of characteristic parameters in viable, aged and non-viable seeds. Std.: standard; Sig.: significance.

Variate	Seed Viability Type (I)	Seed Viability Type (J)	Mean Difference (I-J)	Std. Error	Sig.	95% Confidence Interval	
						Lower Bound	Upper Bound
*rT_drop_*	Viable	Aged	0.088575000	0.051886357	0.096	−0.01655674	0.19370674
		Non-viable	−0.073350000	0.059913204	0.229	−0.19474568	0.04804568
	Aged	Viable	−0.088575000	0.051886357	0.096	−0.19370674	0.01655674
		Non-viable	−0.161925000 *	0.051886357	0.003	−0.26705674	−0.05679326
	Non-viable	Viable	0.073350000	0.059913204	0.229	−0.04804568	0.19474568
		Aged	0.161925000 *	0.051886357	0.003	0.05679326	0.26705674
*rT_min_*	Viable	Aged	0.440695000	0.249822823	0.086	−0.06549412	0.94688412
		Non-viable	−0.479800000	0.288470548	0.105	−1.0642969	0.10469685
	Aged	Viable	−0.440695000	0.249822823	0.086	−0.94688412	0.06549412
		Non-viable	−0.920495000 *	0.249822823	0.001	−1.4266841	−0.41430588
	Non-viable	Viable	0.479800000	0.288470548	0.105	−0.10469685	1.06429685
		Aged	0.920495000 *	0.249822823	0.001	0.41430588	1.42668412
*rT*_0*h*_	Viable	Aged	−0.388177271 *	0.131625596	0.005	−0.65487606	−0.12147848
		Non-viable	−0.206019553	0.151988147	0.183	−0.51397679	0.10193768
	Aged	Viable	0.388177271 *	0.131625596	0.005	0.12147848	0.65487606
		Non-viable	0.182157718	0.131625596	0.175	−0.08454107	0.44885651
	Non-viable	Viable	0.206019553	0.151988147	0.183	−0.10193768	0.51397679
		Aged	−0.182157718	0.131625596	0.175	−0.44885651	0.08454107
*rT*_20*h*_	Viable	Aged	0.174533924	0.153147282	0.262	−0.13577194	0.48483979
		Non-viable	−0.298041417	0.176839249	0.100	−0.65635177	0.06026894
	Aged	Viable	−0.174533924	0.153147282	0.262	−0.48483979	0.13577194
		Non-viable	−0.472575341*	0.153147282	0.004	−0.78288121	−0.16226947
	Non-viable	Viable	0.298041417	0.176839249	0.100	−0.06026894	0.65635177
		Aged	0.472575341 *	0.153147282	0.004	0.16226947	0.78288121
*rT*_40*h*_	Viable	Aged	−0.057694319	0.125059184	0.647	−0.31108830	0.19569966
		Non-viable	−0.428964520 *	0.144405907	0.005	−0.72155868	−0.13637036
	Aged	Viable	0.057694319	0.125059184	0.647	−0.19569966	0.31108830
		Non-viable	−0.371270201 *	0.125059184	0.005	−0.62466418	−0.11787622
	Non-viable	Viable	0.428964520 *	0.144405907	0.005	0.13637036	0.72155868
		Aged	0.371270201 *	0.125059184	0.005	0.11787622	0.62466418
*rT*_60*h*_	Viable	Aged	−0.141687776	0.124322859	0.262	−0.39358982	0.11021426
		Non-viable	−0.449798325 *	0.143555672	0.003	−0.74066975	−0.15892690
	Aged	Viable	0.141687776	0.124322859	0.262	−0.11021426	0.39358982
		Non-viable	−0.308110548 *	0.124322859	0.018	−0.56001259	−0.05620851
	Non-viable	Viable	0.449798325 *	0.143555672	0.003	0.15892690	0.74066975
		Aged	0.308110548 *	0.124322859	0.018	0.05620851	0.56001259

* The mean difference is significant at the 0.05 level.

**Table 4:** The results of cross validation of the SVM model with the whole germination temperature data.

Definition	Fold 1	Fold 2	Fold 3	Fold 4	Fold 5
Training number	96	96	96	96	96
Prediction number	24	24	24	24	24
Misjudgment number/Prediction number in Viable seed (Accuracy rate)	1/9 (88.89%)	0/8 (100%)	0/8 (100%)	0/8 (100%)	2/8 (75%)
Misjudgment number/Prediction number in Aged seed (Accuracy rate)	1/7 (85.71%)	1/8 (87.5%)	1/8 (87.5%)	0/8 (100%)	0/1 (100%)
Misjudgment number/Prediction number in Non-viable seed (Accuracy rate)	0/8 (100%)	0/8 (100%)	0/8 (100%)	0/8 (100%)	0/15 (100%)
Accuracy rate	91.67%	95.83%	95.83%	100%	91.67%
Accuracy rate of total	95%				

**Table 5:** Classification results of SVM model with the whole germination temperature data.

Definition	Viable Type	Aged Type	Non-Viable Type
Prediction number	41	32	47
Classification in Viable seed	38	1	0
Classification in Aged seed	1	29	0
Classification in Non-viable seed	2	2	47
Misjudgment number/Prediction number	3/41	3/32	0/47
Accuracy rate	92.68%	90.63%	100%
Accuracy rate of total	95%		

**Table 6:** The results of cross validation of the SVM model with the first three hours temperature data.

Definition	Fold 1	Fold 2	Fold 3	Fold 4	Fold 5
Training number	96	96	96	96	96
Prediction number	24	24	24	24	24
Misjudgment number/Prediction number in Viable seed (Accuracy rate)	1/9 (88.89%)	0/8 (100%)	0/8 (100%)	0/8 (100%)	0/8 (100%)
Misjudgment number/Prediction number in Aged seed (Accuracy rate)	2/7 (71.43%)	2/8 (75%)	4/8 (50%)	0/8 (100%)	0/1 (100%)
Misjudgment number/Prediction number in Non-viable seed (Accuracy rate)	0/8 (100%)	0/8 (100%)	0/8 (100%)	0/8 (100%)	1/15 (93.33%)
Accuracy rate	87.5%	91.67%	83.33%	100%	95.83%
Accuracy rate of total	91.67%				

**Table 7:** Classification results of SVM method with the first three hours temperature data.

Definition	Viable Type	Aged Type	Non-Viable Type
Prediction number	41	32	47
Classification in Viable seed	40	4	0
Classification in Aged seed	0	24	1
Classification in Non-viable seed	1	4	47
Misjudgment number/Prediction number	1/41	8/32	1/47
Accuracy rate	97.56%	75%	97.87%
Accuracy rate of total	91.67%		
